# Retraction: Dysregulation of circulating miRNAs promotes the pathogenesis of diabetes-induced cardiomyopathy

**DOI:** 10.1371/journal.pone.0296476

**Published:** 2023-12-22

**Authors:** The PLOS ONE Editors

The *PLOS ONE* Editors retract this article [1] because it was identified as one of a series of submissions for which we have concerns about competing interests and peer review. We regret that the issues were not addressed prior to the article’s publication.

The *PLOS ONE* Editors note that primary data were not provided with the article, contrary to the Data Availability statement.

All authors either did not respond directly or could not be reached.
